# A high performance prediction of HPV genotypes by Chaos game representation and singular value decomposition

**DOI:** 10.1186/s12859-015-0493-4

**Published:** 2015-03-05

**Authors:** Watcharaporn Tanchotsrinon, Chidchanok Lursinsap, Yong Poovorawan

**Affiliations:** 1 Advanced Virtual and Intelligent Computing Research Center (AVIC), Department of Mathematics and Computer Science, Faculty of Science, Chulalongkorn University, Phayathai Road, Bangkok, Thailand; 2 Center of Excellence in Clinical Virology, Department of Pediatrics, Faculty of Medicine, Chulalongkorn University, Phayathai Road, Bangkok, Thailand

**Keywords:** HPV, Genotype, Chaos game representation, Singular value decomposition, Prediction

## Abstract

**Background:**

Human Papillomavirus (HPV) genotyping is an important approach to fight cervical cancer due to the relevant information regarding risk stratification for diagnosis and the better understanding of the relationship of HPV with carcinogenesis. This paper proposed two new feature extraction techniques, i.e. ChaosCentroid and ChaosFrequency, for predicting HPV genotypes associated with the cancer. The additional diversified 12 HPV genotypes, i.e. types 6, 11, 16, 18, 31, 33, 35, 45, 52, 53, 58, and 66, were studied in this paper.

In our proposed techniques, a partitioned Chaos Game Representation (CGR) is deployed to represent HPV genomes. ChaosCentroid captures the structure of sequences in terms of centroid of each sub-region with Euclidean distances among the centroids and the center of CGR as the relations of all sub-regions. ChaosFrequency extracts the statistical distribution of mono-, di-, or higher order nucleotides along HPV genomes and forms a matrix of frequency of dots in each sub-region. For performance evaluation, four different types of classifiers, i.e. Multi-layer Perceptron, Radial Basis Function, K-Nearest Neighbor, and Fuzzy K-Nearest Neighbor Techniques were deployed, and our best results from each classifier were compared with the NCBI genotyping tool.

**Results:**

The experimental results obtained by four different classifiers are in the same trend. ChaosCentroid gave considerably higher performance than ChaosFrequency when the input length is one but it was moderately lower than ChaosFrequency when the input length is two. Both proposed techniques yielded almost or exactly the best performance when the input length is more than three. But there is no significance between our proposed techniques and the comparative alignment method.

**Conclusions:**

Our proposed alignment-free and scale-independent method can successfully transform HPV genomes with 7,000 - 10,000 base pairs into features of 1 - 11 dimensions. This signifies that our ChaosCentroid and ChaosFrequency can be served as the effective feature extraction techniques for predicting the HPV genotypes.

**Electronic supplementary material:**

The online version of this article (doi:10.1186/s12859-015-0493-4) contains supplementary material, which is available to authorized users.

## Background

Human Papillomavirus (HPV) is a small double-stranded and most common sexually transmitting DNA virus. At present, more than one hundred types of Human papillomavirus have been identified. They are differentiated by the genetic sequence of the outer capsid protein L1. Approximately forty types can infect the mucosal epithelium. They are categorized according to their epidemiologic association with cervical cancer. Infection with low risk HPV types such as types 6 and 11 can cause benign or low-grade cervical cell abnormalities and genital warts. In contrast, high risk HPV types such as 16 and 18 act as carcinogens that can lead to the development of cervical cancer and other anogenital cancers.

Cervical cancer is the second most common cancer significantly causing morbidity and mortality in women worldwide [1]. Persistent infection by high risk HPV is a necessary cause of this cancer. Especially, the most common high risk HPV types are 16 and 18, and approximately 70% of cervical cancer is due to infection by these genotypes [2]. Each genotype of HPV has a different risk level in the cervical cancer. Furthermore, there is a wide variation in genotype distribution in different regions around the world. To better understand the relationship of HPV with carcinogenesis, many countries have investigated the HPV infection among women with cytological status by HPV genotyping methods, as revealed in Switzerland [3], in Italy [4], in Cambodia [5], and in Romania [6].

HPV genotyping is necessary for managing effective medical treatment strategies to patients with persistent infection and for evaluating prevention strategies to individual patients to be immunized with type-specific HPV vaccines [7]. Currently, there are various kinds of HPV genotyping tests used for detecting the genotypes of Human Papillomavirus, in clinical laboratories. For example, *PapilloCheck*®;, *PCR-RFLP*, *HPV genome sequencing*, *INNO-LiPA*, *Linear* *Array*®; *HPV Genotyping Test*, etc. These methods detect the HPV genotypes from some regions of genomes. Even though these HPV genotyping tests are beneficial and employed for HPV diagnosis in patients nowadays, they have some limitations. To illustrate this aspect, the HPV genotypes are hardly detected in cases of inadequate samples or low amplification signals of some genotypes. Contamination with previously amplified material can lead to false positive results. Furthermore, mistaken classifications can be occurred through cross-reactivity among similar types in the tests based on hybridization [8].

To avoid these problems, some computational methods for identifying HPV types were developed [9-16]. Since discriminating whether the patients have been infected with the high risk types of Human papillomavirus is the most important and urgent aspect for diagnosis and treatment, multiple perspectives were proposed to focus on predicting the HPV risk types. For instance, Wang and Xiao [9] presented multitudinous physicochemical and statistical features from the protein sequences using Fuzzy K nearest neighbor classifier for the risk type prediction of Human papillomaviruses. They also further developed the better algorithm based on geometric moments of protein distance matrix images using a Fuzzy K nearest neighbor classifier [10]. In addition, classification of HPV risk types was also proposed through algorithms based on decision tree [11], text mining [12], genetic mining of DNA sequence structures [13], support vector machines [14], gap-spectrum kernels [15], and ensemble support vector machines with protein secondary structures [16].

While classifying the HPV into high and low risk types is the urgent aspect for diagnosis of the cancer as claimed by many researchers, the study on how to predict specific genotypes of the virus has not significantly focused. In fact, the identification of HPV genotypes infecting the patients is more essential than a rough classification of HPV risk types. To clarify this issue, HPV genotyping can provide more information regarding risk stratification. With the persistent infection, the risk of a precancerous lesion is in between 10% to 15% with HPV types 16 and 18 but below 3% for all other high risk types combined [2]. Furthermore, the relevant diagnosis with cost effectiveness can be done by selecting the virus types to be tested based on epidemiological and prevalence studies from a wide variation in the genotype distribution in different regions around the world. The diversity of virus types and the incidence of multiple infections have made it necessary to develop reliable methods to identify the different genotypes for epidemiological studies and medical treatment. HPV genotyping can make a great contribution to the following aspects: HPV diagnosis in case of single and multiple infection, more information regarding risk stratification, a better understanding of the relationship of HPV with carcinogenesis, and prevention of the cancer though the development of type-specific vaccines. Consequently, HPV genotyping has become an important approach to fight with cervical cancer. For these reasons, this research concentrated on the prediction of HPV genotypes.

Chaos Game Representation (CGR) was proposed as a unique and scale-independent representation for genomic sequences by Jeffrey [17]. It is an iterative mapping technique assigning each nucleotides in a DNA or amino acids in a protein to a unique coordinates in a 2-dimensional space. It can be viewed as a 2-dimensional image of distributed dots and captured in a form of 0-1 square matrix, where 1 represents a dot and 0 represents an empty coordinate. The distribution of positions has two properties of uniqueness and possibility to inverse a coordinate back to its corresponding nucleotide or amino acid [18]. Using graphic approaches to study biological systems can provide useful intuitive insights, as indicated by many previous studies on a series of important biological topics, such as DNA [19,20], RNA [21], genome [22-26], protein [27-35], drug metabolism systems [36], protein-protein interactions [37], analysis of protein sequence evolution [38]. Moreover, the cellular automaton graph has also been applied to study hepatitis B viral infections, HBV virus gene missense mutation, as well as represent complicated biological sequences and help to identify various protein attributes [39-41].

Singular value decomposition (SVD) is a matrix factorization technique with various applications. For instance, it can be used to solve underdetermined and overdetermined systems of linear equations, find inverse and the pseudo-inverse matrices, compute the matrix condition number and calculate the vector system orthogonality and orthogonal complement [42]. SVD is also applied to several areas in gene expression data and microarray data, such as analysis [43-46], search [47], image compression [48], gene extraction [42], and classification [49,50], etc. In this paper, we deployed SVD a tool to reduce the size of CGR into a smaller number of feature matrices without losing any knowledge from the original data. Therefore, a new feature extraction was proposed based on the combination of chaos game representation and singular value decomposition.

Due to the significance of HPV genotyping, the objective of this paper is to predict the HPV genotypes from their genomes, which is similar to the conventional methods of genome detection in clinical laboratories. The remaining sections of this paper are organized as follows. Section “Methods” describes for the methods used in this experiment, including collection of HPV data set, the proposed feature extraction techniques, predicting systems, and performance evaluation. Section “Results and discussion” illustrates the experimental results and discussion. Section “Conclusion” concludes the paper.

## Methods

As realized by a series recent publications [51-58] in response to the call from [59], the following procedures to establish a really useful statistical predictor for a biological system were involved in our method: (i) construct or select valid benchmark data sets to train and test the predictor; (ii) formulate the biological samples with an effective mathematical expression that can truly reflect their intrinsic correlation with the predicted target; (iii) introduce or develop a powerful predicting algorithm (or engine); (iv) properly perform cross-validation tests to objectively evaluate the anticipated accuracy of the predictor; (v) establish a user-friendly web-server for the predictor accessible to the public. The detail of each procedure is discussed as follows.

HPV genome data from genotypes were collected and their features were extracted by our proposed feature extraction techniques, i.e. ChaosCentroid and ChaosFrequency, as inputs for classification. These features were divided into the training and testing sets by a 2-fold cross validation technique. Four different classification models were deployed to train and test the experimental data sets. Then, the prediction performance from the obtained results were evaluated and compared with other methods. Our proposed method consists of the following four main procedures, i.e. data collection, feature extraction, prediction, and performance evaluation.

### Collection of HPV data set

To remove the homologous sequences from the benchmark data sets, a cut-off threshold of 25% was imposed in [60,61] to exclude those proteins from the benchmark data sets that are equal to or greater than 25% of sequence identity to any others in a same subset. However, in this study we did not use such a stringent criterion because the currently available data do not allow us to do so. Otherwise, the numbers of genomes for some subsets would be too few to have statistical significance.

HPV genotypes collected in this experiment are those important genotypes detectable by *Linear* *Array*®; *HPV Genotyping Test*. This HPV genotyping is a widely used qualitative test developed by Roche Molecular Diagnostics for detecting HPV genotypes associated with cervical cancer. The test can detect 37 high and low risk HPV genotypes, including those considered as a significant risk factor for HSIL progression to cervical cancer. To challenge the prediction, only HPV genotypes having genome diversity were concentrated in this experiment. Some of 37 genotypes containing few genomes were excluded. For this reason, only HPV genotypes 6, 11, 16, 18, 31, 33, 35, 45, 52, 53, 58 and 66 were involved. The genomes of these HPV genotypes were collected from the National Center for Biotechnology Information (http://www.ncbi.nlm.nih.gov/). The data set contains Human Papillomavirus genomes of 12 genotypes, including high, possible high, and low risk types. For each HPV genotype, the number of genomes as well as the minimum and maximum lengths are shown in Table 1.

**Table 1:** **The number of genomes, minimum and maximum genome lengths of HPV genotypes in the HPV data set**

**HPV Genotypes**	**No. of genomes**	**Genome length (base pairs)**	
		**Minimum**	**Maximum**
6	58	7954	8051
11	49	7931	10424
16	103	7881	7976
18	19	7824	7857
31	23	7878	7945
33	22	7830	7912
35	28	7820	7908
45	12	7841	7858
52	22	7933	7974
53	16	7856	7863
58	37	7814	7836
66	11	7816	7824

All viral genomes in this HPV data set were previously published and are publicly available on GenBank or NCBI databases. In addition, the genome names, NCBI access numbers, and HPV genotypes of all genomes in the HPV data set are properly cited in Additional file 1, and the HPV data set used in this experiment is also available in Additional file 2.

### Detail of proposed feature extraction techniques

The following techniques, i.e. ChaosCentroid and ChaosFrequency, were proposed to extract the features from the chaos game representation of HPV genomes. To identify each genotype, the relations among subsets of HPV genomes must be clarified. These relations are actually the local features. Since the CGR captures the information of the whole genome data, extracting the global features from the CGR may not be efficient enough to distinguish the HPV genotypes. The local features hidden in various sub-regions of CGR must be more contemplated. In this work, we concentrate on extracting the local features rather than global features. The difference between ChaosCentroid and ChaosFrequency are the feature representation. HPV genomes contain A, C, G, and T nucleotides. Prior to the discussion of ChaosCentroid and ChaosFrequency, the detail of how to construct CGR is the following. Let *x*_*i*_ and *y*_*i*_ be the coordinates of nucleotide *η*_*i*_ at the *i* ^*th*^ position in the nucleotide sequence. Algorithm 1 illustrates how to construct a CGR for capturing a given nucleotide sequence.

**Figure 1:**
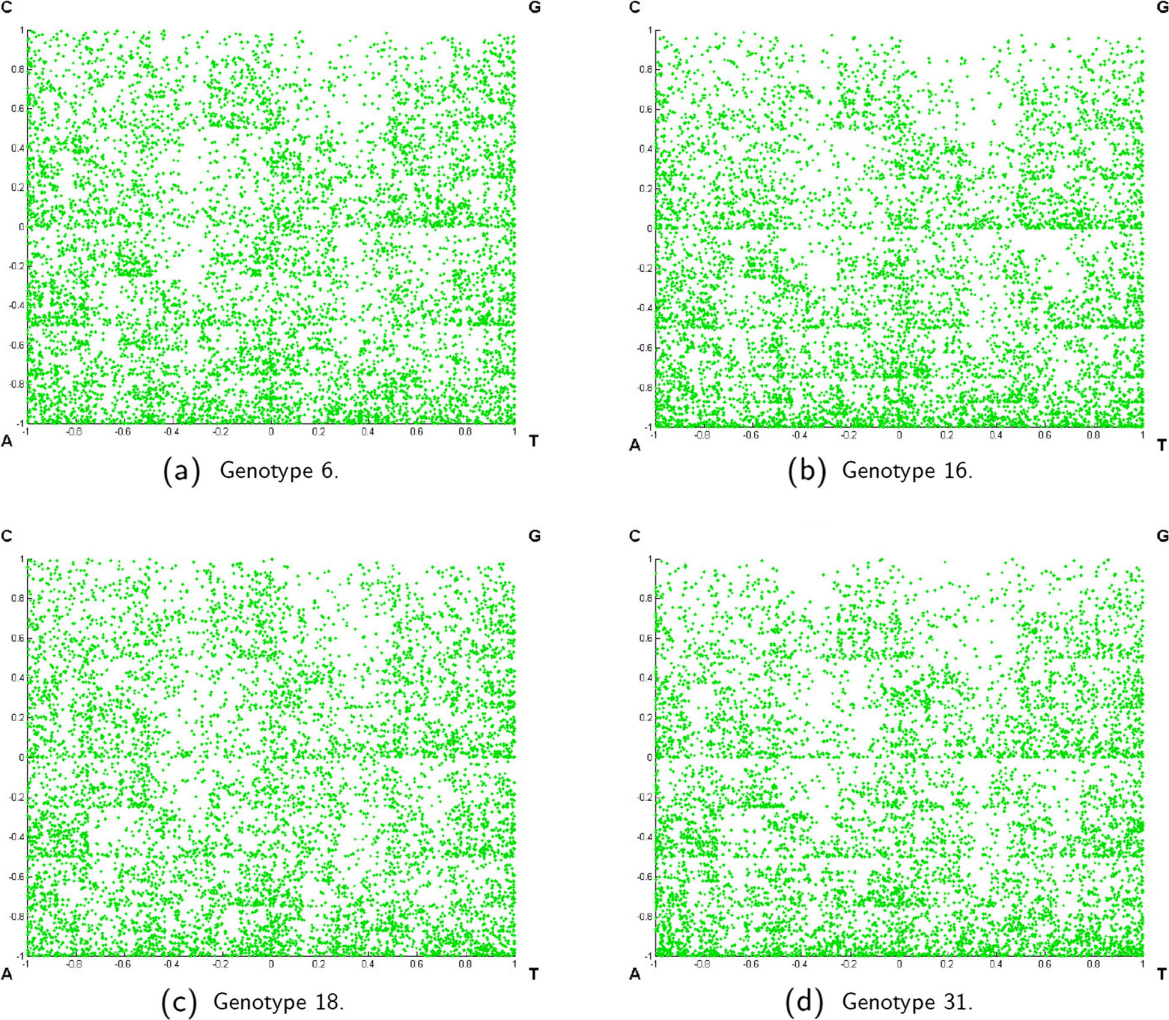
**Chaos game representation (CGR) of HPV genotypes 6, 16, 18, and 31.** **(a)** Genotype 6. **(b)** Genotype 16. **(c)** Genotype 18. **(d)** Genotype 31.



A CGR can be viewed as a square whose corners are at coordinates (-1,-1), (-1,1), (1,1), and (1,-1) representing nucleotides A, C, G, and T, respectively. Note that the size of CGR according to the coordinates of A, C, G, and T nucleotides is equal to 2×2 units. However, this unit size of original CGR is not appropriate for discussing our algorithm. Therefore, the geometrical structure and the physical size of our CGR are re-defined as follows. The size of CGR square is set to *n*×*n* and *n*∈*R* ^+^. Its center is also located at the coordinates (0,0). Each corner of this square represents the same nucleotide as that of the original CGR. After Algorithm 1, CGR can be viewed as an image of distributed dots. Figure 1 shows some examples of CGR of HPV genotypes 6, 16, 18, and 31. Obviously, the number of dots in a CGR is equal to the number of nucleotides in a given sequence. Although this CGR image can be directly used in the prediction step, its computational time may be too high due to the large number of dots. Thus it is necessary to extract only those relevant features from this set of dots to reduce the computational time complexity in the prediction process. In this paper, we proposed two different features as the representation of CGR image. The first feature is called *ChaosCentroid* and the second one is called *ChaosFrequency*. The detail of each feature is the following.

#### ChaosCentroid

**Figure 2:**
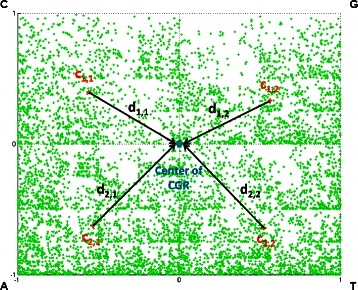
**The distances between the centroids and the center of CGR for HPV genotype 16 after being partitioned into sub-regions of size 2×2.**



According to [17], the *k*-th dot plotted on the CGR of a sequence corresponds to the first *k*-long initial sub-sequence of the sequence. Therefore, any visible pattern of the CGR corresponds to some pattern of the nucleotide sequence. CGR represents the global information of the nucleotide sequence. Partitioning the CGR into several sub-regions is implemented for revealing local information of the interested areas. If two dots are within the same quadrant, they correspond to sequences with the same last mononucleotide; if they are in the same sub-quadrant, the sequences have the same last dinucleotides; and so on. This can demonstrate the structure of the sequences yielding the dots. ChaosCentroid utilizes this biological significance by computing the centroid of the distributed dots of each sub-region. Therefore, the centroid, which can be converted to specific structure of the sequence, is represented as local information of the sub-region. For ChaosCentroid, the CGR is partitioned into $\frac {n}{g} \times \frac {n}{g}$ equal sub-regions, where $\frac {n}{g} \in \{1,2,3,\ldots,11\}$. This range is obtained by all possible numbers that can applied to the CGR. For instance, the CGR is not partitioned when $\frac {n}{g} = 1$, the CGR is partitioned into 4 equal sub-regions when $\frac {n}{g} = 2$, and so on. Furthermore, if the value of $\frac {n}{g}$ is greater than 11, some sub-regions does not contain any dots. So, 11 is the maximum value of $\frac {n}{g}$ in this experiment. For each of $\frac {n}{g}$ partitioned into the CGR, the centroid of each sub-region is computed first. Then all pairs of distances between the centroids and the center of CGR are computed and captured in a form of a matrix. This set of distances can be considered as the relation of information embedded in all sub-regions. However, the number of ChaosCentroids may be too large. Therefore, this matrix is decomposed by applying singular value decomposition (SVD) method to reduce information complexity. Finally, the $\frac {n}{g}$ diagonal elements from the $\frac {n}{g}$-by-$\frac {n}{g}$ diagonal matrix of SVD are represented as the features of CGR and are subsequently used as the input vectors for prediction process. As a result, ChaosCentroid produces 11 formats of input vectors, i.e. the first format have 1 dimension, the second format have 2 dimensions, and so on. Extracting ChaosCentroid consists of the following steps, as illustrated in Algorithm 2. Additionally, Figure 2 shows an example of distances between the centroid of each sub-region and the center of CGR for HPV genotype 16 after being partitioned into sub-regions of size 2×2.

#### ChaosFrequency

As elucidated in [20], the bias of distribution of different mono-, di-, tri-, or higher order nucleotides along the DNA/RNA sequences can generate different patterns in the CGR. This can be used as diagnostic patterns for different HPV genotypes. The CGRs of the HPV genomes of different genotypes tend to exhibit distinct patterns visually, as displayed in Figure 1. Thus, ChaosFrequency concentrates on the frequencies of sub-sequences occurred in the HPV genomes. Particularly, when $\frac {n}{g}$ is equal to 2^*k*^ where *k*∈{1,2,3}, it represents the k-mer frequency occurred in the HPV sequences. Accordingly, the ratio between the number of dots in the sub-region and the total number of dots in the CGR are computed and represented as the feature of each sub-region. This ratio can be interpreted as the *probability of distribution*. Suppose each sub-region is of size *g*×*g*. After extracting the ChaosFrequency of each sub-region, the whole CGR be viewed as a matrix of size $\frac {n}{g} \times \frac {n}{g}$. This matrix is decomposed by SVD to extract the $\frac {n}{g}$ diagonal elements used as the feature of CGR. Likewise, this technique produces 11 formats of input vectors, in accordance with those of ChaosCentroid. The detail of this procedure is illustrated in Algorithm 3. Each sub-region is referred by its location according to the row and column after the partition of CGR. Let *m*_*i*,*j*_ be the number of dots in sub-region at row *i* and column *j*. Suppose there are total *M* dots in CGR. Then we can calculate the probability of distribution as $p_{i,j}=\frac {m_{i,j}}{M}$.



### Predicting systems

To evaluate the performance of the proposed feature extraction techniques, the testing sets were fed to four different types of predicting systems. Each system has its own principle and criteria for predicting the corresponding HPV genotypes. The predicting systems are multi-layer perceptron neural network, radial basis function network, k-nearest neighbor technique, and fuzzy k-nearest neighbor technique. From 400 HPV genomes, one of 12 genotypes which are types 6, 11, 16, 18, 31, 33, 35, 45, 52, 53, 58, and 66 was identified. The detail of set-up for each predicting system in our experiments are as follows.

#### Multi-layer perceptron neural network

Each input pattern is the feature vector **F** obtained from Algorithms 2 and 3. Therefore, the numbers of input neurons are ranged from 1 to 11 according to the sizes of the feature vector **F**. The number of hidden neurons was empirically varied from 1 to 24 neurons to find the most suitable number. From the experiments, 16 hidden neurons are the best number of neurons for producing the best prediction of HPV genotypes. There are 12 output neurons, each of which corresponds to each HPV genotype. To make the testing efficient, the neuron 1 is for determining HPV genotype 6; neuron 2 for type 11; neuron 3 for type 16; neuron 4 for type 18; neuron 5 for type 31; neuron 6 for type 33; neuron 7 for type 35; neuron 8 for type 45; neuron 9 for type 52; neuron 10 for type 53; neuron 11 for type 58; and neuron 12 for type 66. Therefore, the network deployed in our experiments consists of an input layer with $\frac {n}{g}$ neurons, a hidden layer with 16 neurons, and an output layers with 12 neurons. Backpropagation learning rule was adopted to adjust the weights of the network during the training process. Mean squared normalized error function was used as a terminating criterion in the training process. In testing procedure, the predict HPV genotype is determined by this equation. Let *o*_*i*_ be the output value of output neuron *i*.

(1) $$ \text{HPV genotype} = \text{argtype} \max_{1 \leq i \leq 12} (o_{i})  $$

*argtype* is the mapping from neuron index to its corresponding HPV genotype previously defined.

#### Radial basis function network

After finding the optimal spread distances for the prediction, the spread of radial basis function (RBF) is set to 0.4 for ChaosCentroid and 0.1 for ChaosFrequency. The same network structure of multi-layer perceptron was adopted for this RBF network. The determination in Equation (1) of HPV genotypes for multi-layer perceptron was used in this RBF predicting system.

#### K-nearest neighbor technique

In this technique, the determination of HPV genotypes depends upon the value of *k* nearest neighbors measured by Euclidean distance. For any tested feature vector, the HPV genotype of its nearest neighbor is assigned as the HPV genotype of the tested feature vector. Empirically, it was found that *k*=1 gave the best performance.

#### Fuzzy K-nearest neighbor technique

Fuzzy k-nearest neighbor technique was proposed by James M. Keller, Michael R. Gray, and James A. Givens [62]. It is a special variation of the k-nearest neighbor technique family. The algorithm of fuzzy k-nearest neighbor assigns class membership to a sample vector rather than assigning the vector to a particular class. An advantage is that no arbitrary assignments are made by the algorithm. Additionally, membership values of the vector should provide a level of assurance to accompany the resultant classification. In this technique, we set *k* to 1.

### Performance evaluation

Among the independent statistical accuracy testing methods for predicted results such as sub-sampling (e.g., 2, 5 or 10-fold cross-validation) test and jackknife test, jackknife test was deemed the most objective that can always yield a unique result for a given benchmark data set, as elucidated in [59] and demonstrated by Equations 28, 29 and 30 in [59]. Therefore, the jackknife test has been increasingly used and widely recognized by investigators to test the power of various prediction methods (see, e.g., [63-73]). Although jackknife is widely used, its computational time is rather high. However, to reduce the computational time, we adopted the 2-fold cross-validation in this experiment to deal with the parameter optimization. Therefore, the reported prediction performance was obtained by the combination of both validating sets.

In this experiment, we adopted Equation 11 of [52] to formulate the set of four metrics, including Sensitivity(Sen), Specificity(Spec), Accuracy(Acc), and Matthew’s Correlation Coefficient(MCC), for evaluating the prediction performance. The formulation of the four metrics is defined by the following equations.

(2) $$ {\fontsize{8}{12}\begin{aligned} &{}Sensitivity = 1-\frac{N^{+}_{-}}{N^{+}}, \qquad\qquad\qquad\quad\quad\;\;0 \leq Sen \leq 1 \\ &{}Specificity = 1-\frac{N^{-}_{+}}{N^{-}}, \qquad\qquad\qquad\quad\quad0 \leq Spec \leq 1 \\ &{}Accuracy = 1-\frac{N^{+}_{-}+N^{-}_{+}}{N^{+}+N^{-}}, \,\,\,\,\quad\qquad\quad\qquad0 \leq Acc \leq 1 \\ &{}MCC \,=\, \frac{1\!-\left(\frac{N^{+}_{-}}{N^{+}}+\frac{N^{-}_{+}}{N^{-}}\right)} {\sqrt{\left(1+\frac{N^{-}_{+}-N^{+}_{-}}{N^{+}}\right) \left(1+\frac{N^{+}_{-}-N^{-}_{+}}{N^{-}}\right)}}, \,\qquad\,-1\! \!\leq \!MCC\! \leq \!1 \end{aligned}}  $$

where *N* ^+^ is the total number of HPV genomes of the investigated genotype whereas $N^{+}_{-}$ the number of HPV genomes of the investigated genotype that is incorrectly predicted as the other genotypes; *N* ^−^ the total number of HPV genomes of the other genotypes that are not investigated whereas $N^{-}_{+}$ the number of HPV genomes of the other genotypes that is incorrectly predicted as the investigated genotype. The investigated HPV genotype is 6, 11, 16, 18, 31, 33, 35, 45, 52, 53, 58, or 66. For example, if the investigated genotype is 6, *N* ^+^ is the total number of HPV genomes of genotype 6, while *N* ^−^ is the total number of the genomes of the other genotypes, excluding genotype 6.

According to Equation 2, the prediction performance can be evaluated in a meaningful explanation, as follows. The sensitivity is used for evaluating the performance of the predicting systems in identifying the investigated genotype. When $N^{+}_{-} = 0$, none of HPV genomes of the investigated genotype was incorrectly predicted as the other genotypes, so the sensitivity is 1. In contrast, while $N^{+}_{-} = N^{+}$, all HPV genomes of the investigated genotype were incorrectly predicted as the other genotypes, so the sensitivity is 0. The specificity is used for evaluating the performance of the systems in excluding the other genotypes. When $N^{-}_{+} = 0$, none of HPV genomes of the other genotypes was incorrectly predicted as the investigated genotype, so the specificity is 1; while $N^{-}_{+} = N^{-}$, all HPV genomes of the other genotype were incorrectly predicted as the investigated genotype, so the specificity is 0. The accuracy is used for evaluating the performance of the systems in classifying the investigated genotype and the other genotypes. When $N^{+}_{-} = N^{-}_{+} = 0$, none of HPV genomes of the investigated genotype and none of HPV genomes of the other genotypes was incorrectly predicted, so the accuracy is 1; while $N^{+}_{-} = N^{+}$ and $N^{-}_{+} = N^{-}$ all HPV genomes of the investigated genotype and all HPV genomes of the other genotypes were incorrectly predicted, so the accuracy is 0. Typically, the Matthew’s Correlation Coefficient (MCC) is used for measuring the quality of binary classification. When $N^{+}_{-} = N^{-}_{+} = 0$, none of HPV genomes of the investigated genotypes and none of HPV genomes of the other genotypes was incorrectly predicted, so MCC is 1; when $N^{+}_{-} = N^{+}/2$ and $N^{-}_{+} = N^{-}/2$, MCC is 0 meaning no better than random prediction; When $N^{+}_{-} = N^{+}$ and $N^{-}_{+} = N^{-}$, MCC is -1 indicating total disagreement between prediction and observation.

However, the set of metrics in Equation 2 is valid only for single-label systems. For multi-label systems whose existence has become more frequent in system biology [61,74] and system medicine [67,75], a completely different set of metrics as defined in [76] is needed.

## Results and discussion

The value of variable $\frac {n}{g}$ in Algorithms 2 and 3 was set from 1 to 11. The performance of HPV genotype prediction was separately summarized according to each predicting system and two feature extracting schemes. The obtained results are the following.

### Results from multi-layer perceptron neural network

The results of the HPV genotype prediction gained by ChaosCentroid and by ChaosFrequency feature extraction with the predicting system based on multi-layer perceptron neural network are summarized in Tables 7 and 8, respectively, of Additional file 3. The results were reported according to different values of $\frac {n}{g} \in \{1, 2, \ldots, 11\}$. It is rather remarkable when $\frac {n}{g} = 1$.

When $\frac {n}{g} = 1$, the number of sub-regions of CGR is equal to one. Thus there is only one centroid computed by ChaosCentroid and the probability of distribution of CGR computed by ChaosFrequency is equal to one. The overall performance of ChaosFrequency is much lower than those of ChaosCentroid. ChaosFrequency gain 0*%* of sensitivity and 100*%* of specificity in all genotypes, excepting genotype 16. It implies that the features of all genomes extracted by ChaosFrequency are totally predicted to genotype 16. In contrast, ChaosCentroid can obtain high performance metrics, including accuracy, sensitivity, specificity, and Matthew’s Correlation Coefficient in almost all genotypes. This is because a centroid is computed from the coordinates of every dots. It is obvious that different HPV genotypes must have different distribution of dots and centroids. So, predicting HPV genotypes with high performance from these centroids is possible. But in case of ChaosFrequency, the probability of distribution of every HPV genotype is equal. This makes the feature of each HPV genotype indistinguishable.

However, when the value of $\frac {n}{g}$ is greater than one, the local information regarding the frequency of sub-sequence among nucleotides in each sub-region is brought out and the performance is increased in proportion to the value of $\frac {n}{g}$. It is noticeable that there is no significant difference between the overall performance obtained from ChaosCentroid and ChaosFrequency when $\frac {n}{g} > 3$. In addition, we can conclude that, to achieve high performance of prediction, the local information of each sub-region is more relevant than global information.

### Results from radial basis function network

The results of the HPV genotype prediction gained by ChaosCentroid and by ChaosFrequency feature extraction with the predicting system based on radial basis function network are summarized in Tables 9 and 10, respectively, of Additional file 3. According to the results, the performance values obtained by this predicting system are unstable among input dimensions. This is because this experiment set only one optimal spread distance, which gain the maximum average accuracy of all dimensions, for each predicting system of ChaosCentroid and ChaosFrequency, respectively. In fact, it is possible that each input dimension has its own proper spread distance, and one value of spread distance can not fit for all dimensions. In addition, it is noticeable that ChaosFrequency with RBF at 4-dimensional input can achieve the best performance with minimum input dimension. The overall performance trend obtained from this predicting system is similar to those of multi-layer perceptron. But the peformance from multi-layer perceptron is significantly higher than the performance from radial basis function.

### Results from K-nearest neighbor technique

The results of the HPV genotype prediction gained by ChaosCentroid and by ChaosFrequency feature extraction with the predicting system based on k-nearest neighbor technique are summarized in Tables 11 and 12, respectively, of Additional file 3. The experimental results have shown the high performance of prediction. Therefore, it can imply that, in each sub-region, the structure of sequence in a form of centroid by ChaosCentroid and the statistical distribution of mono-, di-, or higher order nucleotides in a form of frequency by ChaosFrequency, are closed to each other in the same genotype. The overall performance trend obtained from this predicting system is similar to those of multi-layer perceptron. But the performance from this predicting system is slightly higher than the performance of multi-layer perceptron.

### Results from Fuzzy K-nearest Neighbor Technique

The results of the HPV genotype prediction gained by ChaosCentroid and by ChaosFrequency feature extraction with the predicting system based on fuzzy k-nearest neighbor technique are summarized in Tables 13 and 14, respectively, of Additional file 3. The overall performance trend obtained from this predicting system is similar to those of multi-layer perceptron. Additionally, the overall performance of this predicting system is slightly higher than the performance of multi-layer perceptron but it is statistically equal to the performance of k-nearest neighbor technique due to setting the same value of *k*.

### Comparative results with Related Method

NCBI viral genotyping tool [77] is a web-based tool for identifying the genotype of a viral sequence. It works by sliding a window along the query sequence and processing each window/sequence segment separately. Each segment is compared to a set of reference sequences using BLAST, which returns the similarity scores for the local alignments. The reference sequence genotype that matches the query with the highest similarity score is assigned to the query segment. The process is repeated for the next window until the whole length of the query sequence has been covered. The results from all windows are combined. If the same genotype is assigned to most segments, then the query sequence is considered the genotype. This tool is a web-based resource that provides a reliable method based on alignment. Then, this experiment adopted this tool for identifying genotypes of the viral genomes in the HPV data set. To evaluate the prediction performance, the result obtained by this genotyping tool were compared with the best results obtained by the proposed ChaosCentroid and ChaosFrequency feature extraction techniques with all predicting systems, as illustrated in Tables 2, 3, 4, 5 and 6.

**Table 2:** **Best results of the HPV genotype prediction based on the features extracted by ChaosCentroid and by ChaosFrequency with multi-layer perceptron neural network**

**HPV Genotypes**	**ChaosCentroid**				**ChaosFrequency**			
	**Accuracy**	**Sensitivity**	**Specificity**	**MCC**	**Accuracy**	**Sensitivity**	**Specificity**	**MCC**
6	100.00	100.00	100.00	1.00	100.00	100.00	100.00	1.00
11	100.00	100.00	100.00	1.00	100.00	100.00	100.00	1.00
16	100.00	100.00	100.00	1.00	100.00	100.00	100.00	1.00
18	100.00	100.00	100.00	1.00	100.00	100.00	100.00	1.00
31	100.00	100.00	100.00	1.00	100.00	100.00	100.00	1.00
33	100.00	100.00	100.00	1.00	100.00	100.00	100.00	1.00
35	100.00	100.00	100.00	1.00	100.00	100.00	100.00	1.00
45	100.00	100.00	100.00	1.00	100.00	100.00	100.00	1.00
52	100.00	100.00	100.00	1.00	100.00	100.00	100.00	1.00
53	100.00	100.00	100.00	1.00	100.00	100.00	100.00	1.00
58	100.00	100.00	100.00	1.00	100.00	100.00	100.00	1.00
66	100.00	100.00	100.00	1.00	100.00	100.00	100.00	1.00

**Table 3:** **Best results of the HPV genotype prediction based on the features extracted by ChaosCentroid and by ChaosFrequency with radial basis function network**

**HPV Genotypes**	**ChaosCentroid**				**ChaosFrequency**			
	**Accuracy**	**Sensitivity**	**Specificity**	**MCC**	**Accuracy**	**Sensitivity**	**Specificity**	**MCC**
6	100.00	100.00	100.00	1.00	100.00	100.00	100.00	1.00
11	100.00	100.00	100.00	1.00	100.00	100.00	100.00	1.00
16	99.50	99.03	99.66	0.99	100.00	100.00	100.00	1.00
18	100.00	100.00	100.00	1.00	100.00	100.00	100.00	1.00
31	100.00	100.00	100.00	1.00	100.00	100.00	100.00	1.00
33	100.00	100.00	100.00	1.00	100.00	100.00	100.00	1.00
35	99.50	96.43	99.73	0.96	100.00	100.00	100.00	1.00
45	100.00	100.00	100.00	1.00	100.00	100.00	100.00	1.00
52	100.00	100.00	100.00	1.00	100.00	100.00	100.00	1.00
53	100.00	100.00	100.00	1.00	100.00	100.00	100.00	1.00
58	100.00	100.00	100.00	1.00	100.00	100.00	100.00	1.00
66	100.00	100.00	100.00	1.00	100.00	100.00	100.00	1.00

**Table 4:** **Best results of the HPV genotype prediction based on the features extracted by ChaosCentroid and by ChaosFrequency with k-nearest neighbor technique**

**HPV Genotypes**	**ChaosCentroid**				**ChaosFrequency**			
	**Accuracy**	**Sensitivity**	**Specificity**	**MCC**	**Accuracy**	**Sensitivity**	**Specificity**	**MCC**
6	100.00	100.00	100.00	1.00	100.00	100.00	100.00	1.00
11	100.00	100.00	100.00	1.00	100.00	100.00	100.00	1.00
16	100.00	100.00	100.00	1.00	100.00	100.00	100.00	1.00
18	100.00	100.00	100.00	1.00	100.00	100.00	100.00	1.00
31	100.00	100.00	100.00	1.00	100.00	100.00	100.00	1.00
33	100.00	100.00	100.00	1.00	100.00	100.00	100.00	1.00
35	100.00	100.00	100.00	1.00	100.00	100.00	100.00	1.00
45	100.00	100.00	100.00	1.00	100.00	100.00	100.00	1.00
52	100.00	100.00	100.00	1.00	100.00	100.00	100.00	1.00
53	100.00	100.00	100.00	1.00	100.00	100.00	100.00	1.00
58	100.00	100.00	100.00	1.00	100.00	100.00	100.00	1.00
66	100.00	100.00	100.00	1.00	100.00	100.00	100.00	1.00

**Table 5:** **Best results of the HPV genotype prediction based on the features extracted by ChaosCentroid and by ChaosFrequency with fuzzy k-nearest neighbor technique**

**HPV Genotypes**	**ChaosCentroid**				**ChaosFrequency**			
	**Accuracy**	**Sensitivity**	**Specificity**	**MCC**	**Accuracy**	**Sensitivity**	**Specificity**	**MCC**
6	100.00	100.00	100.00	1.00	100.00	100.00	100.00	1.00
11	100.00	100.00	100.00	1.00	100.00	100.00	100.00	1.00
16	100.00	100.00	100.00	1.00	100.00	100.00	100.00	1.00
18	100.00	100.00	100.00	1.00	100.00	100.00	100.00	1.00
31	100.00	100.00	100.00	1.00	100.00	100.00	100.00	1.00
33	100.00	100.00	100.00	1.00	100.00	100.00	100.00	1.00
35	100.00	100.00	100.00	1.00	100.00	100.00	100.00	1.00
45	100.00	100.00	100.00	1.00	100.00	100.00	100.00	1.00
52	100.00	100.00	100.00	1.00	100.00	100.00	100.00	1.00
53	100.00	100.00	100.00	1.00	100.00	100.00	100.00	1.00
58	100.00	100.00	100.00	1.00	100.00	100.00	100.00	1.00
66	100.00	100.00	100.00	1.00	100.00	100.00	100.00	1.00

**Table 6:** **Results of the HPV genotype prediction obtained by NCBI viral genotyping tool**

**HPV Genotypes**	**Accuracy**	**Sensitivity**	**Specificity**	**MCC**
6	100.00	100.00	100.00	1.00
11	100.00	100.00	100.00	1.00
16	100.00	100.00	100.00	1.00
18	100.00	100.00	100.00	1.00
31	100.00	100.00	100.00	1.00
33	100.00	100.00	100.00	1.00
35	100.00	100.00	100.00	1.00
45	100.00	100.00	100.00	1.00
52	100.00	100.00	100.00	1.00
53	100.00	100.00	100.00	1.00
58	100.00	100.00	100.00	1.00
66	100.00	100.00	100.00	1.00

The experimental results have shown that all methods, excepting ChaosCentroid with radial basis function network, can achieve the best performance of the four metrics, including accuracy, sensitivity, specificity, and Matthew’s Correlation Coefficient, in predicting the HPV genotypes of the data set. It demonstrated that both of the proposed techniques and the NCBI genotyping tool can be used to predict the genotypes of HPV genomes. Even though there is no significance between the proposed techniques and the NCBI genotyping tool, some issues should be considered.

The NCBI genotyping tool provides a reliable method based on homology searching sequence alignment procedure. The limitation of alignment is that it is difficult to identify or classify the protein or DNA sequences in the case that they does not have a significant sequence homology. Besides, the alignment with multiple sequences will take time consuming and only one query sequence at a time can be processed by this tool. So, this method is not appropriate for large scale tasks.

In contrast, the proposed techniques, i.e. ChaosCentroid and ChaosFrequency, are based on Chaos game representation, which provides a unique and scale-independent representation of DNA sequences through the statistical distribution of mono-, di-, tri-, or higher order nucleotides along DNA sequences. An advantage of CGR over alignment is that it has the potential to reveal the evolutionary and/or functional relationships between the sequences having no significant homology, as elucidated in [35]. Furthermore, it does not require prior knowledge of consensus sequences, nor does it involve exhaustive searches for sequences in databases. The limitation of CGR is that it takes a computational time to generate the representations from DNA sequences. Nevertheless, this experiment utilized the singular value decomposition to reduce the size of CGR into a smaller number of feature matrices so the computational time in the prediction process was also reduced. From the experimental results, it have shown that the proposed ChaosCentroid and ChaosFrequency, which are based on chaos game representation and singular value decomposition, can successfully extract the characteristic parameters of HPV genotypes for the prediction.

Since user-friendly and publicly accessible web-servers represent the future direction for developing practically more useful models, simulated methods, or predictors [78-80], we may make efforts in our future work to provide a web-server for the method presented in this paper.

## Conclusion

This paper proposed two new feature extraction techniques, i.e. ChaosCentroid and ChaosFrequency, based on chaos game representation and singular value decomposition for predicting HPV genotypes from nucleotide sequences in HPV genomes. Both extracting techniques concentrate on the local information among nucleotides. For the sub-regions in CGR, ChaosCentroid pays attention to capture the structures of the sequences in a form of centroids, while ChaosFrequency focuses on capture the distribution of sub-sequences in a form of frequencies. Four different predicting systems, i.e. multi-layer perceptron neural network, radial basis function network, K-nearest neighbor technique, and fuzzy K-nearest neighbor technique, were deployed. From the experiment, we found that the features extracted by our proposed feature extraction techniques are significant and independent of the predicting systems. The comparative results demonstrated no significance between our proposed techniques and the NCBI viral genotyping tool. In addition, local information is more important than global information in order to achieve high performance of prediction.

## Additional files

Additional file 1
**Genome names, NCBI access numbers, and HPV genotypes of all genomes in the HPV data set.**

Additional file 2
**HPV data set.**

Additional file 3
**Results of the HPV genotype prediction based on the features extracted by ChaosCentroid and ChaosFrequency with all predicting systems.**

## Abbreviations

HPV: Human papillomavirus
LSIL: Low grade squamous intraepithelial lesion
HSIL: High grade squamous intraepithelial lesion
CGR: Chaos game representation
SVD: Singular value decomposition
MLP: Multi-layer perceptrons
RBF: Radial basis function
KNN: K-Nearest neighbor
FKNN: Fuzzy K-Nearest neighbor
